# The Novel Direct AR Target Gene *Annexin A2* Mediates Androgen-Induced Cellular Senescence in Prostate Cancer Cells

**DOI:** 10.1007/s10528-024-10953-9

**Published:** 2024-11-19

**Authors:** Kimia Mirzakhani, Mehdi Heidari Horestani, Julia Kallenbach, Golnaz Atri Roozbahani, Aria Baniahmad

**Affiliations:** Institute of Human Genetics, Jena University Hospital, Am Klinikum 1, 07740 Jena, Germany

**Keywords:** Prostate cancer, Bipolar androgen therapy, HSP27, Cellular senescence

## Abstract

Clinical trials for prostate cancer (PCa) patients have implemented the bipolar androgen therapy (BAT) that includes the treatment with supraphysiological androgen level (SAL). SAL treatment induces cellular senescence in tumor samples of PCa patients and in various PCa cell lines, including castration-resistant PCa (CRPC), and is associated with enhanced phospho-AKT levels. Using an AKT inhibitor (AKTi), the SAL-mediated cell senescence is inhibited. Here, we show by RNA-seq analyses of two human PCa cell lines, that annexin A2 (*ANXA2*) expression is induced by SAL and repressed by co-treatment with AKTi. Higher *ANXA2* expression is associated with better survival of PCa patients and suggests that ANXA2 is part of SAL-mediated tumor suppressive activity. ChIP-seq revealed that AR is recruited to the intronic regions of *ANXA2* gene suggesting that *ANXA2* is a novel direct AR target gene. Knockdown of ANXA2 shows that SAL-induced cellular senescence is mediated by ANXA2 and enhances the levels of phospho-AKT indicating an interaction between the AR, ANXA2 and AKT. Notably, we found that the level of heat shock protein HSP27, known to interact with ANXA2, is associated with cellular senescence. HSP27 level is induced by SAL but the induction is blunted by knockdown of ANXA2 suggesting a novel ANXA2-HSP27 pathway in PCa. This was confirmed using an HSP27 inhibitor that reduced the SAL-induced cellular senescence levels suggesting that ANXA2 upregulates HSP27 to mediate AR-signaling in SAL-induced cellular senescence. Thus, the data indicate ANXA2-HSP27 cross-talk as novel factors in the signaling by the AR-AKT pathway to mediate cellular senescence.

**Supplementary Information:**

The online version contains supplementary material available at 10.1007/s10528-024-10953-9.

## Introduction

Prostate cancer (PCa) is diagnosed highest among new incidence cancer in men in US (Siegel et al. 2023). The growth of the normal prostate tissue and progression of PCa relies on the activity of the androgen receptor (AR, *NR3C4*) (Vickman et al. 2020). Accordingly, the inhibition of the AR activity by androgen deprivation therapy and the use of AR antagonists for maximal blockade of AR is used regularly to hormonally treat PCa patients.

Paradoxically, higher concentrations of androgens at supraphysiological androgen level (SAL) also inhibit PCa tumor growth in cell lines and in mouse xenograft model systems. Interestingly, in both androgen dependent and castration-resistant PCa cell lines as well as in PCa samples from patients treated ex vivo, SAL induces cellular senescence (Roediger et al. 2014; Pungsrinont et al. 2020; Kokal et al. 2020; Mirzakhani et al. 2022). SAL is currently used in clinical trials for treatment of metastatic PCa patients by the so-called bipolar androgen therapy (BAT) (Denmeade 2018; Isaacs et al. 2019; Denmeade et al. 2021; Kumar et al. 2023; Markowski et al. 2024).

Consistently, low doses of androgens (defined as 1 pM R1881, LAL) promote growth whereas SAL (defined as 1 nM R1881) inhibits potently PCa cell proliferation and induces cellular senescence, which is dependent on the presence of AR (Roediger et al. 2014; Niu et al. 2008). Mechanistically, SAL leads to increased p-AKT levels and induction of p15^INK4b^ (Mirzakhani et al. 2022). The AKT inhibitor (AKTi) reduced the level of senescent cells in both castration-sensitive (LNCaP) and castration-resistant (C4-2) PCa cell lines induced by SAL (Mirzakhani et al. 2022). This indicates that SAL induces cell senescence in part through the AKT signaling pathway.

Transcriptome analyses revealed that Annexin A2 (ANXA2) is one factor, which is induced by SAL and repressed by AKTi treatment in both PCa cell lines. ANXA2 has many functions including membrane repair, connecting membrane with the cytoskeleton, endo- and exocytosis (Kayejo et al. 2023). Interestingly, higher ANXA2 levels are associated with increased survival of patients with PCa suggesting that treatment with SAL has tumor suppressive activity. Functionally, the knockdown of ANXA2 suggests that it mediates SAL-induced cellular senescence and regulates p-AKT and HSP27 levels. The data suggest that ANXA2 is a direct AR target gene, which is induced by SAL and mediates the induction of cell senescence by SAL. Further, the data suggest that ANXA2 is part of the AR-AKT and HSP27 signaling to regulate the induction of cellular senescence by SAL, thus linking androgen signaling to ANXA2.

## Results

### Transcriptome Analyses Reveal an Upregulation of *ANXA2* by SAL in both LNCaP and C4-2 Cell Lines

**Fig. 1:**
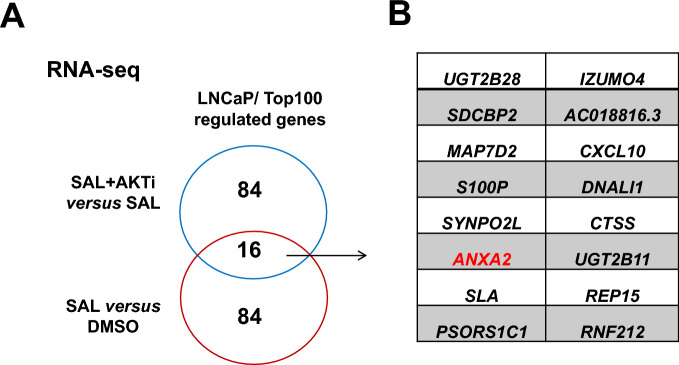
*ANXA2* expression is upregulated by SAL and inhibited by co-treatment with AKTi. Transcriptome analyses using RNA-seq were performed after treatment of cells for 72 h (*n* = 3). **A** Venn diagram indicates the overlap of the top100 significantly SAL-mediated upregulated genes in LNCaP cells and their downregulation by AKTi co-treatment. **B** The overlap of these genes is indicated with highlighted *ANXA2*

RNA-seq data were analyzed for differentially expressed genes to identify the overlap of androgen-mediated upregulated genes comparing upregulation by SAL and downregulation by AKTi. *ANXA2* was identified as one of the 16 genes among the top 100 genes showing the strongest fold change by SAL and downregulation by the co-treatment of SAL with AKTi (Fig. 1A, B).

**Fig. 2:**
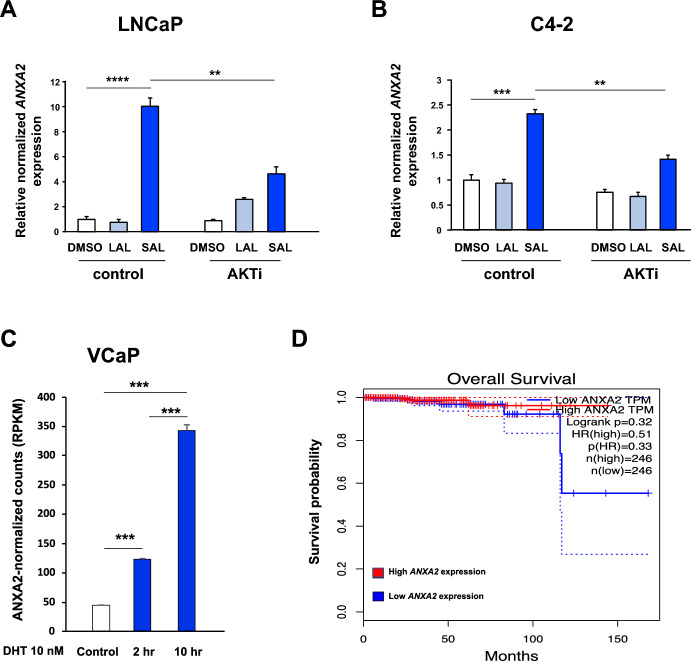
*ANXA2* expression is induced preferentially by SAL in both LNCaP and C4-2 cell lines. To confirm the androgen-mediated regulation of *ANXA2* expression, qRT-PCR experiments were performed with LNCaP (**A**) and C4-2 cells (**B**) treated for 72 h with low androgen level (LAL, 1 pM R1881), supraphysiological androgen level (SAL, 1 nM R1881) and/or AKTi (1 μM). DMSO was used as solvent control. Normalized fold change of mRNA expression is indicated using normalization to the expression of both *TBP* and *GAPDH* as house-keeping genes. (*n* = 3, biological replicates). **C** Expression of *ANXA2* in VCaP cells treated with DHT (10 nM) using RNA-seq data derived from GSE157104 indicated as RPKM (reads per kilo base per million mapped reads). **D** The ANXA2 expression data are plotted against overall survival in months. Data were obtained from the publicly available Gene Expression Profiling Interactive Analysis (GEPIA) database with the indicated hazard ratios (HR) and samples size numbers (*n*). Two-tailed unpaired Student’s *t*-test was performed for statistical analysis (*p ≤ 0.05, **p ≤ 0.01, ***p ≤ 0.001, ****p ≤ 0.0001)

An upregulation of ANXA2 expression by SAL was also observed through transcriptome analyses of RNA-seq data obtained from C4-2 cells (supplemental Fig. S1). To confirm the androgen regulation of gene expression of *ANXA2*, qRT-PCR experiments were performed with both cell lines LNCaP and C4-2. Cells were treated with R1881 at 1 pM for LAL and 1 nM for SAL as defined earlier (Roediger et al. 2014). R1881 is more specific for AR, since dihydrotestosterone (DHT) is metabolized rapidly and the metabolites can act as ligands for the estrogen receptor beta (Handa et al. 2008). DMSO served as the solvent control. These treatments were performed as single treatment or as co-treatment with AKTi (Fig. 2). The results suggest that *ANXA2* mRNA is induced specifically by SAL in both cell lines whereas AKTi inhibits the SAL-mediated induction. The induction of *ANXA2* gene expression was higher in LNCaP (Fig. 2A) compared to C4-2 cells (Fig. 2B).

In line with this, public available RNA-seq data set from GSE157104 (Guo et al. 2021) confirms the induction of *ANXA2* in another human PCa cell line using VCaP cells treated with DHT (Fig. 2C). Moreover, the data suggest a similar tendency but not statistically significant in mouse xenografts using tumor samples from LuCaP patient-derived xenografts (supplemental Fig. S2; Han et al. 2022). Of note, the GEPIA database indicates, although not significantly, that high *ANXA2* mRNA expression is associated with better survival of PCa patients comparing low expression and high expression data plotted against overall survival (Fig. 2D) being in line with previous reports (Ding et al 2010; Grewal et al. 2021) that suggest higher expression level of ANXA2 have tumor suppressive function in PCa.

### SAL Induces the Chromatin Recruitment of AR to *ANXA2* Gene

The induction of gene expression of *ANXA2* by SAL led to the hypothesis that *ANXA2* may be a direct target gene of AR. Chromatin immunoprecipitation experiments with massive parallel sequencing (ChIP-seq) were performed with LNCaP cells with and without SAL treatment.

**Fig. 3:**
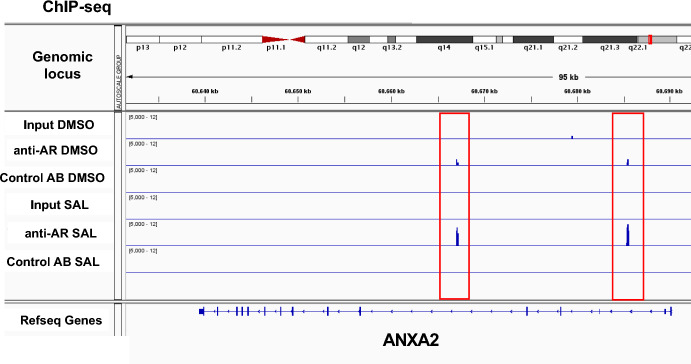
The AR is recruited in a SAL-dependent manner to the *ANXA2* genomic locus. Chromatin immuno-precipitation with subsequent massive parallel sequencing (ChIP-seq) revealed recruitment of AR to the *ANXA2* locus in LNCaP cells. The enrichment of AR is enhanced by SAL treatment at the introns 1 and 3 of the *ANXA2* genomic locus

The data indicate that intronic sequences of ANXA2 are specifically enriched by AR antibody in the presence of SAL in intron 1 and 3 of ANXA2 (Fig. 3). Thus, the data suggest that AR is recruited SAL-dependently to the *ANXA2* gene. Taken together including the RNA-seq data it strongly suggests that *ANXA2* is a novel direct AR target gene.

### ANXA2 Mediates in Part Cellular Senescence Induced by SAL, Functionally Linking AR-AKT to ANXA2 Signaling

**Fig. 4:**
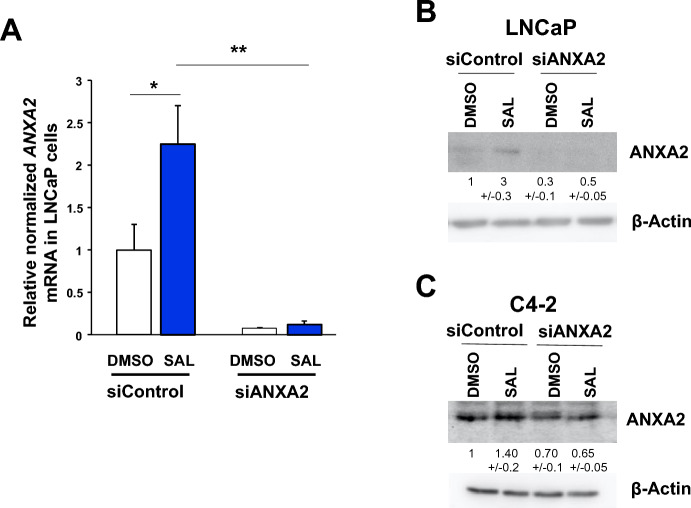
Knockdown of ANXA2 reduces mRNA levels and protein levels upon SAL treatment. LNCaP and C4-2 cells were transiently transfected with siRNA scrambled control or siRNA directed against ANXA2 and treated with DMSO as solvent control or SAL for 72 h. RNA and protein extracts were used for qRT-PCR and Western blot, respectively. **A** Downregulation of *ANXA2* mRNA in cells transfected with siANXA2 (*n* = 3). **B** Reduction of ANXA2 protein levels in LNCaP cells and (**C**) C4-2 cells. Numbers below the immunodetected bands indicate the intensities normalized to β-Actin bands as loading control

To analyze functionally whether ANXA2 is part of the AR-signaling controlled by SAL and whether ANXA2 mediates at least in part cellular senescence induced by SAL, small interfering RNA (siRNA) mediated knockdown experiments were performed directed against *ANXA2*. The knockdown was confirmed at RNA level (Fig. 4A) and protein level (Fig. 4B, C).

**Fig. 5:**
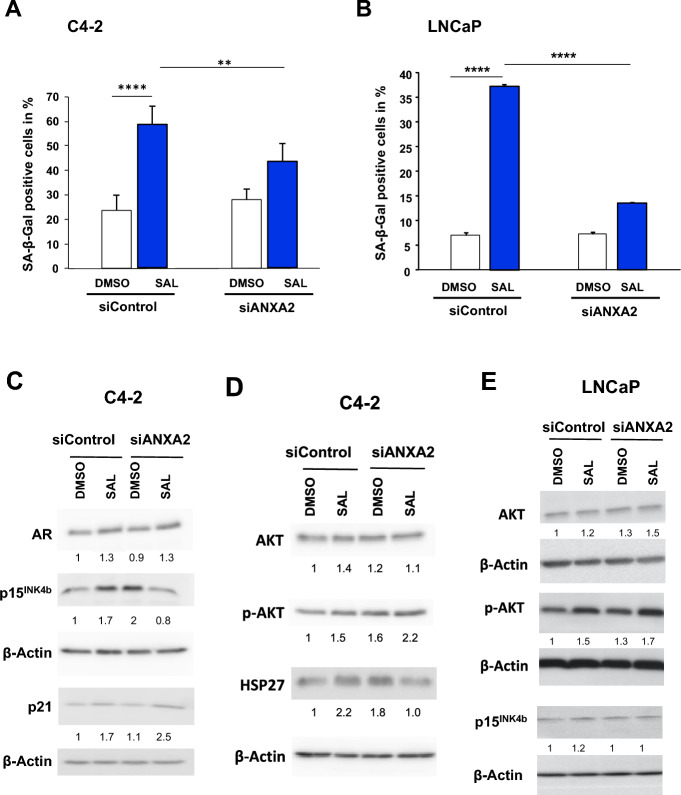
ANXA2 mediates SAL-induced cellular senescence in both LNCaP and C4-2 cell lines. C4-2 and LNCaP cells were transiently transfected with control scrambled siRNA or siANXA2 RNA and treated for 72 h with SAL or DMSO as solvent control. Cells were stained for the senescence-associated β-galactosidase (SA-β-Gal) activity. The percentage of the SA-β-Gal positive cells was calculated in relation to total number of cells per observed field of each transfection and treatment. Three random fields per well were counted and the mean was calculated. **A** C4-2 and **B** LNCaP cells. For each cell line three independent experiments were performed. Student’s t-test was performed for statistical analysis (**p ≤ 0.01, ***p ≤ 0.001, ****p ≤ 0.0001). **C** and **D** Protein levels of AKT, p-AKT, p15^INK4b^, p21 and HSP27 of transiently transfected C4-2 cells with siControl or si*ANXA2*. **E** Protein levels of AKT, p-AKT and p15^INK4b^ of transiently transfected LNCaP cells with siControl or si*ANXA2*. The numbers indicate the protein levels normalized to the loading control β-Actin using LabImage 1D software

As expected, SAL enhances ANXA2 protein level. Similar to data of Fig. 2A, in which the *ANXA2* mRNA induction was stronger in LNCaP cells compared to C4-2 cells, the ANXA2 protein level is more induced by SAL in LNCaP compared to C4-2 cells. The siANXA2-mediated knockdown reduces the protein level in both cell lines (Fig. 4B, C). Analyzing the SAL-mediated induction of cellular senescence, the data suggest that the ANXA2 knockdown inhibits the cellular senescence level specifically after SAL treatment. This was observed in both the castration-resistant C4-2 cell the and androgen-sensitive LNCaP lines (Fig. 5A, B).

Analyzing the levels of the cell cycle inhibitor p15^INK4b^, the data suggest that SAL treatment enhances the protein level whereas siANXA2 blunts this effect, which is in line with the induction of cellular senescence (Fig. 5C-E, supplemental Fig. S3). Expectedly, the AR protein level is stabilized by androgens and seems unaffected by *ANXA2* knockdown. Notably, while the p21 levels are enhanced moderately by SAL as described earlier (Roediger et al. 2014), the knockdown of ANXA2 further enhances p21 levels by SAL treatment (Fig. 5C).

It was reported previously that the lncRNA *SAT1* mediates SAL-induced cellular senescence (Mirzakhani et al. 2022). Interestingly however, the knockdown of ANXA2 did not change the expression of the lncRNA *SAT1* and vice versa (supplemental Fig. S4). This suggests that ANXA2 uses a distinct pathway to mediate SAL-induced cellular senescence and indicates the identification of a novel pathway to induce cellular senescence by SAL.

Taken together, the knockdown of Annexin A2 suggests that the androgen-induced ANXA2 promotes SAL-induced cellular senescence indicating a novel AR-ANXA2 signaling pathway in PCa to induce cellular senescence.

Activated AKT signaling is known as a pro-survival pathway. Since AKT mediates in part the induction of cellular senescence by SAL (Roediger et al. 2014; Mirzakhani et al. 2022), we analyzed AKT and p-AKT levels. The knockdown of *ANXA2* had no measurable change on AKT levels in both cell lines (Fig. 5D, E). Notably, analyzing p-AKT levels, the knockdown of *ANXA2* rather enhances p-AKT level (Fig. 5D, E). This suggests that ANXA2 regulates phosphorylation of AKT and reduces AKT signaling. This suggests that ANXA2 reduces this pro-survival pathway of PCa cells.

It was reported that ANXA2 interacts with the heat shock protein 27 (HSP27) mediating in UVC-resistance in AP1 breast cancer cells (Tong et al. 2008).

**Fig. 6:**
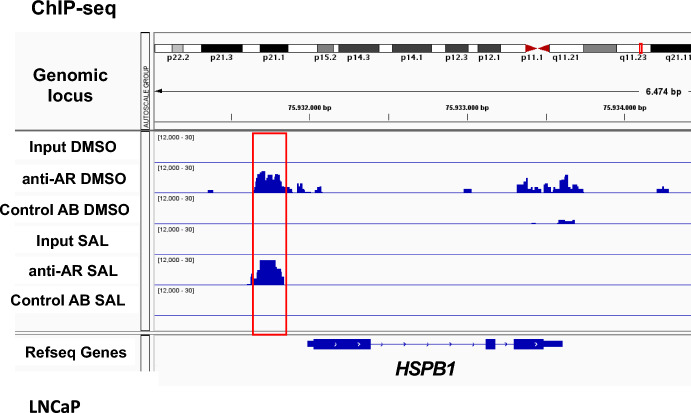
The AR is recruited to the *HSPB1* gene locus encoding HSP27. Chromatin immuno-precipitation with subsequent massive parallel sequencing (ChIP-seq) revealed recruitment of AR to the *HSPB1* locus in LNCaP cells. The enrichment of AR is enhanced at the promoter regions of the *HSPB1* genomic locus. Input and a control antibody (Control AB) were used as background control

Another link between ANXA2 and HSP27 was reported in nasopharyngeal carcinoma in which the knockdown of *ANXA2* activates the HSP27 pathway (He et al. 2022). Therefore, we analyzed the effects of the *ANXA2* knockdown on HSP27 in PCa cells. The inhibition of *ANXA2* expression enhances HSP27 levels confirming that ANXA2 and HPS27 are functionally linked in PCa cells (Fig. 5D). Interestingly, we detected that the HSP27 protein level is induced by SAL (Fig. 5D). RNA-seq data confirmed the upregulation of *HSPB1* mRNA expression, encoding HSP27, by SAL in C4-2 cells (supplementary Fig. S5). The knockdown of *ANXA2* blunts the induction of HSP27 by SAL (Fig. 5D), which is a hint that ANXA2 might enhance HSP27 levels. To address the possibility that the HSP27 encoding gene (*HSPB1*) is regulated by AR, ChIP-seq data from LNCaP cells were analyzed. The data suggest that the AR is recruited to the HSP27 promoter region (Fig. 6). The recruitment of AR is however androgen-independent. Perhaps SAL enhances the recruitment of coactivators to DNA-bound AR.

**Fig. 7:**
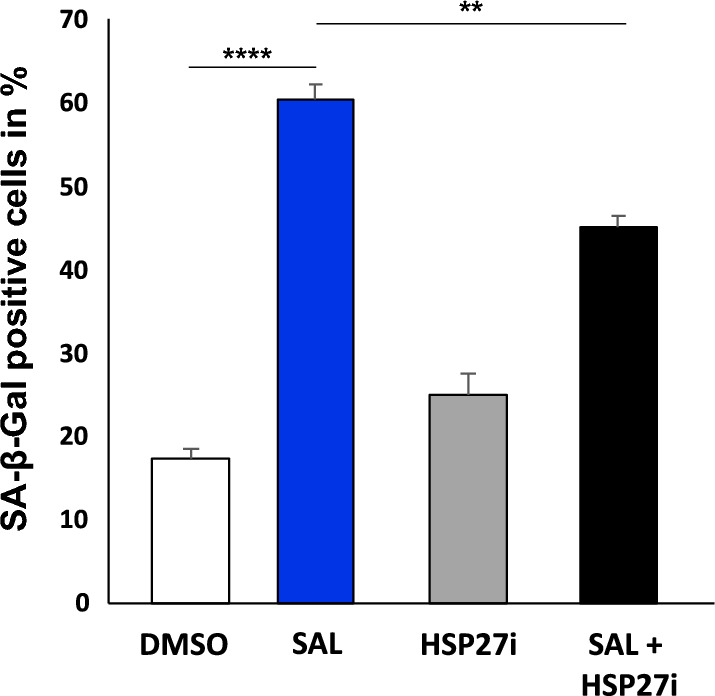
Inhibition of HSP27 activity reduces SAL-induced cellular senescence. Similar experimental setup as described in Fig. 5A using C4-2 cells treated with the HSP27 inhibitor J2 (10 nM) with and without R1881 (1 nM) for 72 h prior the SA-beta galactosidase assay. The percentage of the SA-β-Gal positive cells was calculated in relation to total number of cells per observed field of each transfection and treatment. Three random fields per well were counted and the mean was calculated

Thus, the inhibition of *ANXA2* expression suggests that ANXA2 mediates cellular senescence induced by SAL. The obtained data also suggest that ANXA2 enhances HSP27 levels, however, the induction by SAL treatment is diminished by knockdown of ANXA2 (Fig. 5D). Based on this in order to control cellular senescence by SAL, we hypothesized that induced ANXA2 levels result in enhanced HSP27 activity. Thus, we postulated that the AR- ANXA2- HSP27 signaling axis may regulate cellular senescence by SAL and examined whether HSP27 activity mediates in part the AR-mediated cellular senescence. To address this hypothesis, the HSP27-specific inhibitor J2 was used. Interestingly, although enhancing cellular senescence levels by single treatment, treatment with the HSP27 inhibitor reduces the SAL-induced cell senescence level (Fig. 7).

**Fig. 8:**
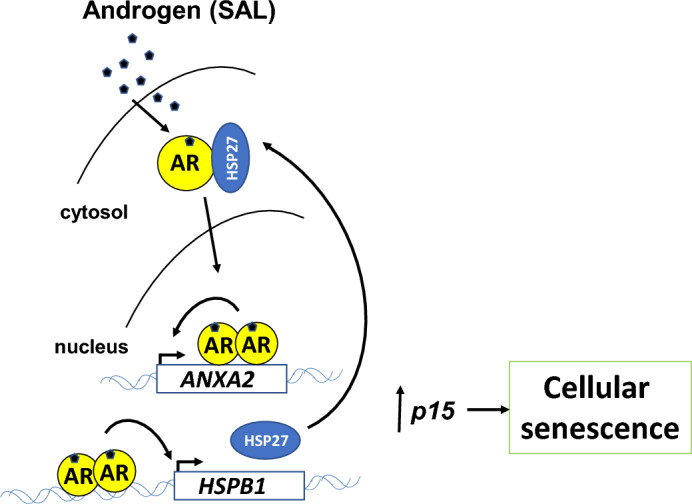
SAL induces cellular senescence through the novel AR-ANXA2-HSP27-p15^INK4b^ pathway. SAL leads to upregulation of both ANXA2 and HSP27 as novel positively regulated AR target genes that mediate cellular senescence by enhancing p15^INK4b^ level in PCa cells

The data suggest that the positively regulated AR target ANXA2 enhances HSP27 and mediates SAL-induced cellular senescence indicating a novel AR-ANXA2-HSP27 axis in bipolar androgen therapy (Fig. 8).

## Discussion and Conclusions

In the bipolar androgen therapy cycling of SAL is used for treatment of metastatic PCa patients. Mechanistically, SAL induces cellular senescence in PCa cells including androgen-sensitive and CRPC cells as well as in human PCa patient-derived samples treated ex vivo with SAL (Roediger et al. 2014; Mirzakhani et al. 2022). The molecular pathway of SAL-induced cellular senescence and cell cycle arrest by AR in PCa is unclear, since treatment with low androgen levels rather promotes growth of PCa cells through the AR. We focused on SAL-induced cellular senescence and identified several factors that are induced by SAL. Besides AKT signaling that mediates in part the SAL-induced cellular senescence (Mirzakhani et al. 2022), also the mitogen-inducible gene 6 (MIG6), as a feedback inhibitor of ERBB-2 that induces mitogenic and transforming activity, was identified recently as a mediator of SAL-induced cellular senescence (Schomann et al. 2022). It seems MIG6 acts as a tumor suppressor in PCa but functions differentially in other cancers. Similarly, ANXA2 has been shown to act functionally in a cancer type and context-dependent manner (Zhang et al., 2012; Grewal et al. 2021; Huang et al. 2022).

In PCa tissue the expression of *ANXA2* is significantly lower compared to samples from patients with benign prostate hyperplasia (Yee et al. 2007; Ding et al. 2010). Further, lower ANXA2 expression is negatively associated with tumor stage and Gleason scores 5–7. *ANXA2* expression decreased steadily with the progression of PCa (Beyene et al., 2018). Also, PCa recurrence and metastasis are associated with lower *ANXA2* expression (Yee et al. 2007), which suggests that ANXA2 is a tumor suppressor in PCa. Accordingly, a higher survival rate of patients with PCa is significantly correlated with higher *ANXA2* expression.

Tumor suppressive association of ANXA2 was also reported for esophageal squamous carcinoma, sinonasal adenocarcinoma, and nasopharyngeal carcinoma. However, a battery of other cancers suggest that ANXA2 has oncogenic properties and shows an upregulation of ANXA2 expression including breast, pancreatic, colorectal cancer, renal cell carcinoma, and acute promyelocytic leukemia (Zhang et al., 2012). Targeting ANXA2 was suggested as a therapeutic approach (Li et al. 2021; Grewal et al. 2021; Gounou et al. 2023). Thus, ANXA2 appears to be involved in a wide range of cellular processes, and its functions may vary depending on the cell type and context in which it is expressed. Since ANXA2 has cancer type dependent activities as a tumor suppressor or oncoprotein a drug targeting system would be useful only for an individualized cancer management (Christensen et al., 2018).

In general, ANXA2, also known as Lipocortin II or Calpactin I, is a member of the annexin A family and located in nucleus, cytoplasm and also at the extracellular cell surface (Zhang et al., 2012). ANXA2 is known to regulate membrane trafficking, to bind to RNA and Ca^2+^ -ions and to interact with many factors including S100A10 forming a heterotetramer which can be translocated upon the activation of the non-receptor tyrosine kinase Src (Bharadwaj et al. 2021; Grewal et al. 2021). Further, it was reported that ANXA2 is part of the cytokinesis of cells for proper mitosis (Benaud et al. 2015).

One of the functions of ANXA2 as an oncoprotein in cancer is to promote invasion and angiogenesis, e.g. by promoting the activation of angiogenic factors such as VEGF (vascular endothelial growth factor). As a tumor suppressor higher ANXA2 levels are associated with better survival of PCa patients. Associated with this, it was reported that in PCa ANXA2 levels are inversely correlated to *ERG* gene expression, known to be a marker for more aggressive PCa (Griner et al., 2015).

Although in some cancer types ANXA2 is associated with activating AKT, the knockdown of ANXA2 reduces AKT protein level in nasopharyngeal carcinoma (Chen et al. 2015), while our data suggest that in PCa cells the knockdown seems not to influence AKT protein levels.

Mechanistically, SAL induces the recruitment of AR to the ANXA2 gene locus and induces its expression indicating that ANXA2 is a direct and positively controlled AR target gene. Inhibition of AKT blunts SAL-mediated induction of *ANXA2* gene expression indicating that the AR-AKT interaction is essential for full ANXA2 induction. Inhibition of AKT reduces also cellular senescence levels mediated by SAL. This led to the hypothesis that ANXA2 mediates in part the induction of cellular senescence mediated by the AR-AKT interaction. In line with this, the inhibition of ANXA2 by knockdown resulted in lower levels of senescent cells when treated with SAL. Thus, the data suggest that ANXA2 as a novel AR target gene induced by SAL mediates SAL-induced cellular senescence in PCa. Of note, this pathway seems to be independent of the previously published AR- lncRNA *SAT1* pathway. Therefore, it indicates the identification of a novel signaling pathway used by the AR.

Interestingly, the knockdown of ANXA2 reduced the protein level of HSP27. HSP27 itself is a client protein of ANXA2 (Tong et al. 2008) and interacts also with AR. Interestingly, in ovarian carcinoma cells the knockdown of HSP27 enhances p21 levels in cytosol linking HSP27 to p21 and to ANXA2 (Lu et al. 2016). Of note, SAL induces HSP27 protein levels, which are associated with induction of cellular senescence. This indicates that the AR-AKT pathway is linked to ANXA2-HSP27 signaling. This led to the hypothesis that HSP27 is responsible for ANXA2 activity to mediate cellular senescence by SAL. In fact, this was observed using the HSP27-specific inhibitor J2. Inhibition of HSP27 reduced the level of cellular senescence induced by SAL. This suggests that for induction of cellular senescence by SAL, a novel pathway is identified consisting of the AR-AKT-ANXA2-HSP27 signaling.

## Experimental Section

### Cell Culture and Treatments

Cell lines, cell culture were described previously (Protopopov et al. 2002; Schomann et al. 2022). Cells were treated for 72 h with 1 pM R1881 (LAL), 1 nM R1881 (SAL), or 0.1% DMSO as shown by Roediger et al. (2014).

RNA-sequencing and transcriptome analysis have been previously described (Mirzakhani et al. 2022). The RNA-seq datasets are available in GEO database. Accession numbers: GSE162711, GSE155528, GSE154755. The GSE179687 was used to analyze ANXA2 expression in mouse xenografts treated with high-T.

ChIP-Seq analysis was performed as described by Schomann et al. (2022). Cells were treated with and without SAL prior chromatin immunoprecipitation (ChIP). DMSO was used as solvent control. Anti-AR antibody from Cell signaling was used for immunoprecipitation after crosslinking and the iDeal ChIP-seq Kit from Diagenode, (Cat.-Nr.: C01010055, Denville, U.S.) was used according to the manufacturer’s protocol including multiple quality controls (Schomann et al. 2022). Input and a control antibody (Control AB) were used as background control. The IGV software was used for visualization.

### Cell Transfection with siRNA

To generate an ANXA2 knockdown with siRNA, C4-2 and LNCaP cells were transfected using Human ANXA2 siRNA SMARTPool (Dharmacon) with a final concentration of 25 nM. As negative control the ON-TARGETplus non-targeting pool was used. In general, the DharmaFECT™ transfection reagent was used according to the manufacturer’s instructions. Briefly, 16 h before transfection 250*10^5^ C4-2 and LNCaP cells were seeded in 6-well cell culture dishes in an antibiotic-free medium. 1 h before transfection, the antibiotic-free medium was refreshed. The siRNAs were diluted in 1 × siRNA buffer and with medium to reach 250 nM final concentration. Diluted siRNAs and siReagent were added to opti-MEM medium separately and kept at room temperature for 5 min. After that, siRNA-opti-MEM was transferred to siReagent-opti-MEM Eppendorf and incubated at room temperature for 20 min. Then 200 µL of the mixture was dropwise added to each respective well. 1 day after transfection, the cells were treated with SAL (1 nM R1881) or 0.1% DMSO as solvent control for 3 days.

### Cellular Senescence Assays

The assays were performed with 6-well plates, and the cells were seeded at 25,000 cells per well. The staining and detection were performed as described previously (Dimri et al. 1995; Esmaeili et al. 2016). At least 3 × 200 cells per well and at least 2 wells per treatment were analyzed to calculate the percentage of SA-β-Gal positive cells.

### Antibodies and Western Blotting

Primary antibodies used for immunodetection include: ANXA2 (Proteintech, 60,051–1-Ig, dilution: 1:1000), panAKT (Cell Signaling, 4685S, dilution: 1:5000), AR (Biogenex, 256 M, 1:2000), β-Actin (Abcam, ab6276, dilution: 1:10,000), p-AKT (S473) (Cell Signaling, 4058S, dilution: 1:5000), p15^INK4b^ (MBiosource, MBS821044, dilution: 1:2000), p21^Cip1^ (Cell Signaling, 2946, dilution: 1:2000), and HSP27 (Enzolifescience, SPA 800D, dilution 1:1000). For detection HRP- IgG (Cell Signaling, 7076S, dilution 1:10,000) or anti-rabbit IgG (Cell Signaling, 7074S, dilution: 1:10,000). Quantifications were performed via the LabImage 1D.

### Quantitative Real Time PCR (qRT-PCR)

Two-step qRT-PCR was performed as described previously (Esmaeili et al. 2016) with gene specific primers. For normalization the housekeeping genes *TBP* and *GAPDH* mRNAs were used.

Primer sequences (5’ → 3’):

**Table:** 

*ANXA2*	fwd rev	GCTCGGGATCTCTATGACGC TACTTTCTGGAGGTGGGGCA
*FKBP5*	fwd rev	GAGGAAACGCCGATGATTGGAGAC CATGCCTTGATGACTTGGCCTTTG
*GAPDH*	fwd rev	AGTCCCTGCCACACTCAG TACTTTATTGATGGTACATGACAAGG
*CDKN2B* (*p15*^*INK4b*^)	fwd rev	GAATGCGCGAGGAGAACAAG TCATCATGACCTGGATCGCG
*TBP*	fwd rev	GGCGTGTGAAGATAACCCAAGG CGCTGGAACTCGTCTCACT

The correlation data for overall survival between ANXA2 expression and PCa patients were retrieved from Gene Expression Profiling Interactive Analysis (GEPIA) datasets (Tang et al. 2017), which provides hazard ratio and log rank.

For statistical analysis the two-tailed unpaired Student’s t-test was used (GraphPad Prism 8.0) A 95% confidence interval (*p*-value *p* < 0.05) was considered as statistically significant (*).

## Supplementary Information

Below is the link to the electronic supplementary material.

Supplementary file1 (PPTX 17204 KB)

## Data Availability

The datasets generated during and/or analyzed during the current study are available from the corresponding author on reasonable request.

## Abbreviations

ANXA2: Annexin A2
AR: Androgen receptor
BAT: Bipolar androgen therapy
ChIP-seq: Chromatin immunoprecipitation sequencing
CRPC: Castration-resistant PCa
HSP: Heat shock protein
LAL: Low androgen level
PCa: Prostate cancer
SAL: Supraphysiological androgen level
SA-β-Gal: Senescence-associated β-galactosidase
siRNA: Small interfering RNA
