# Supplementary material for: The Novel Direct AR Target Gene Annexin A2 Mediates Androgen-Induced Cellular Senescence in Prostate Cancer Cells
Source: Biochem Genet. 2024 Nov 19;63(6):5013–28. doi: 10.1007/s10528-024-10953-9 (PMC12602616; doi:10.1007/s10528-024-10953-9)
Supplement: Supplementary file 1 — Supplementary file1 (PPTX 17204 KB) [file 10528_2024_10953_MOESM1_ESM.pptx]

## Slide 1
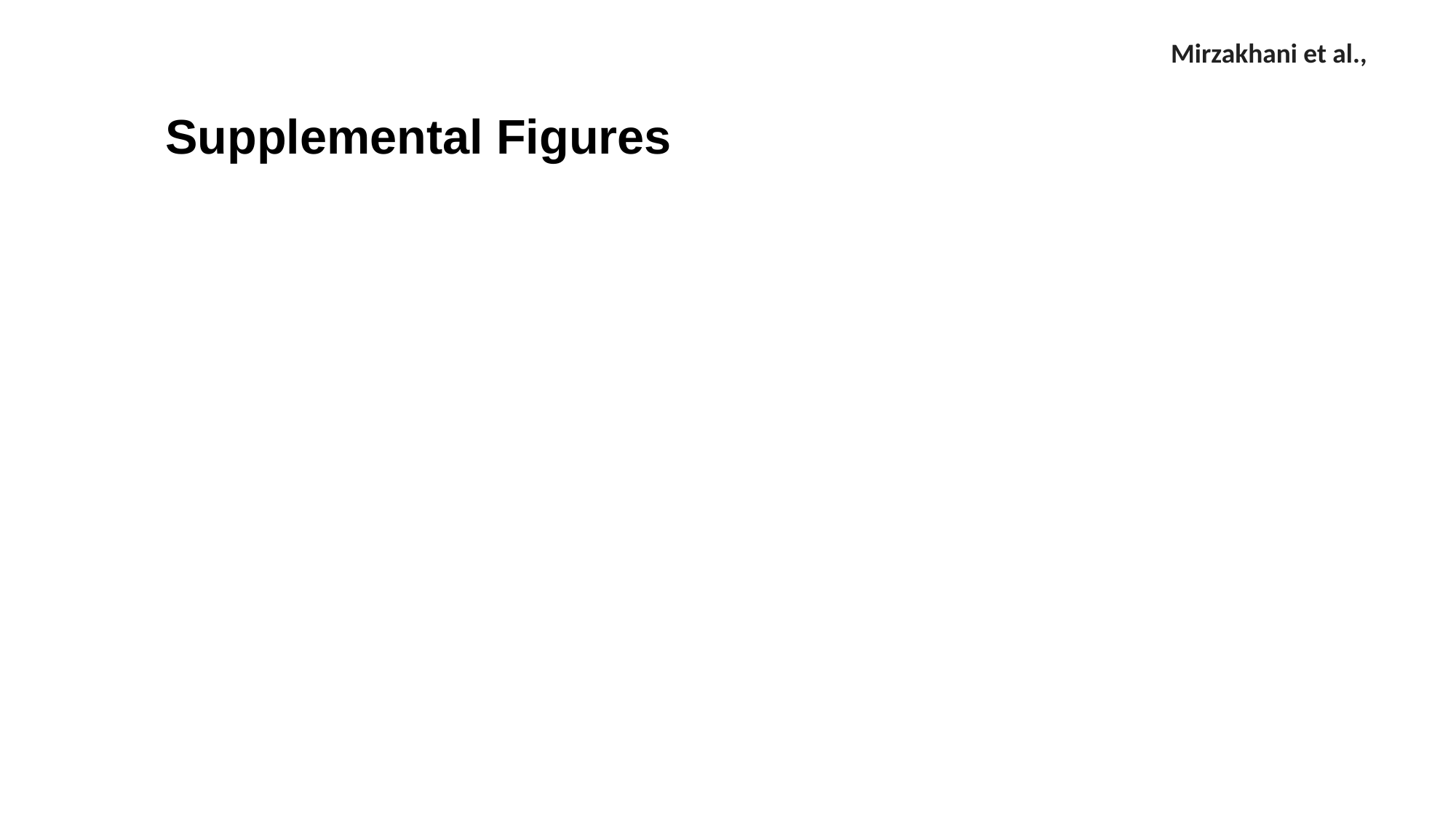

Mirzakhani et al.,
Supplemental Figures

## Slide 2
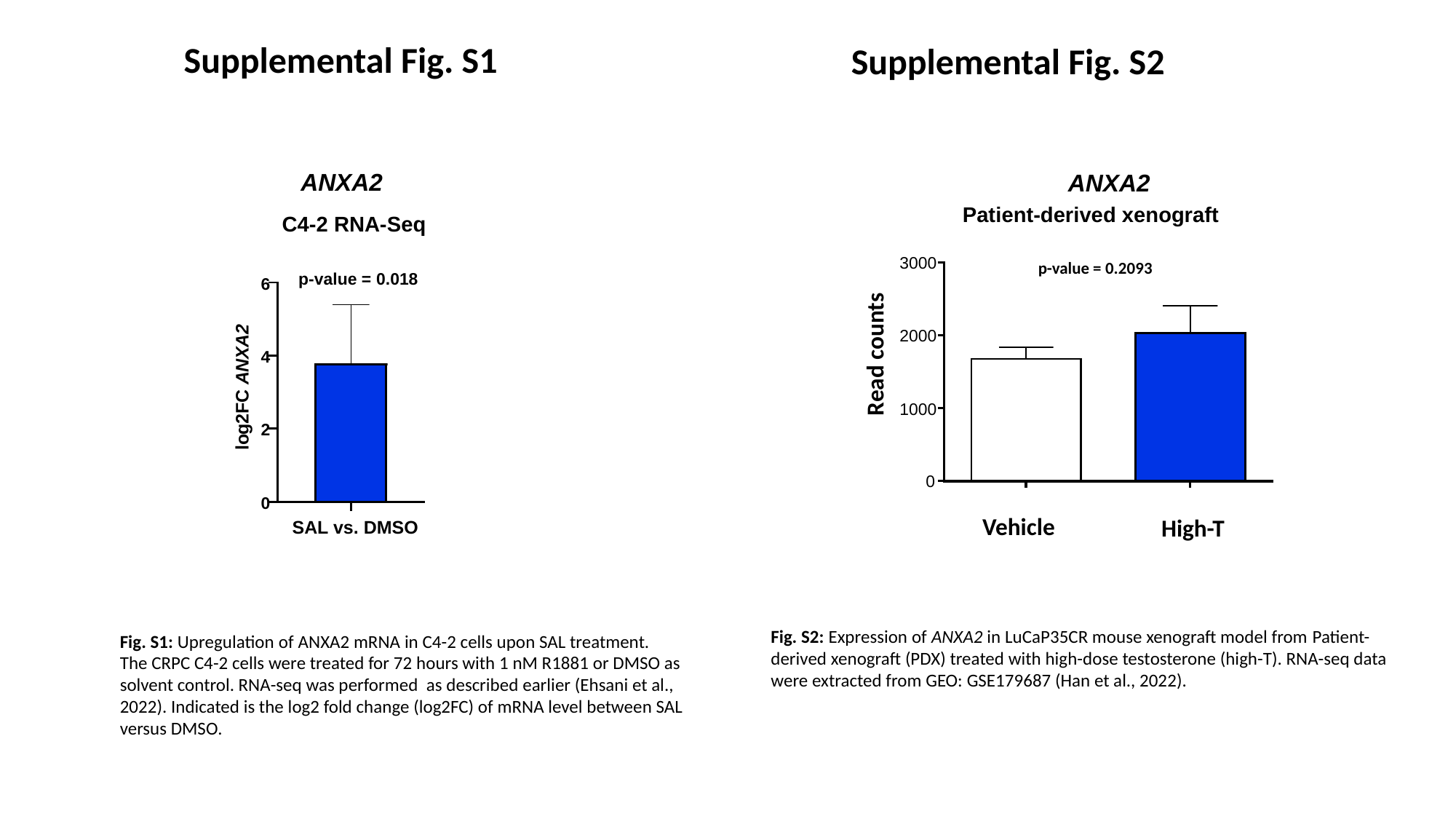

Supplemental Fig. S1
Supplemental Fig. S2
ANXA2
ANXA2
Patient-derived xenograft
C4-2 RNA-Seq
p-value = 0.2093
3000
p-value = 0.018
6
2
2000
A
Read counts
X
4
N
A
C
F
1000
2
g
2
o
l
0
0
Vehicle
High-T
SAL vs. DMSO
Fig. S2: Expression of ANXA2 in LuCaP35CR mouse xenograft model from Patient-derived xenograft (PDX) treated with high-dose testosterone (high-T). RNA-seq data were extracted from GEO: GSE179687 (Han et al., 2022).
Fig. S1: Upregulation of ANXA2 mRNA in C4-2 cells upon SAL treatment.
The CRPC C4-2 cells were treated for 72 hours with 1 nM R1881 or DMSO as solvent control. RNA-seq was performed as described earlier (Ehsani et al., 2022). Indicated is the log2 fold change (log2FC) of mRNA level between SAL versus DMSO.

## Slide 3
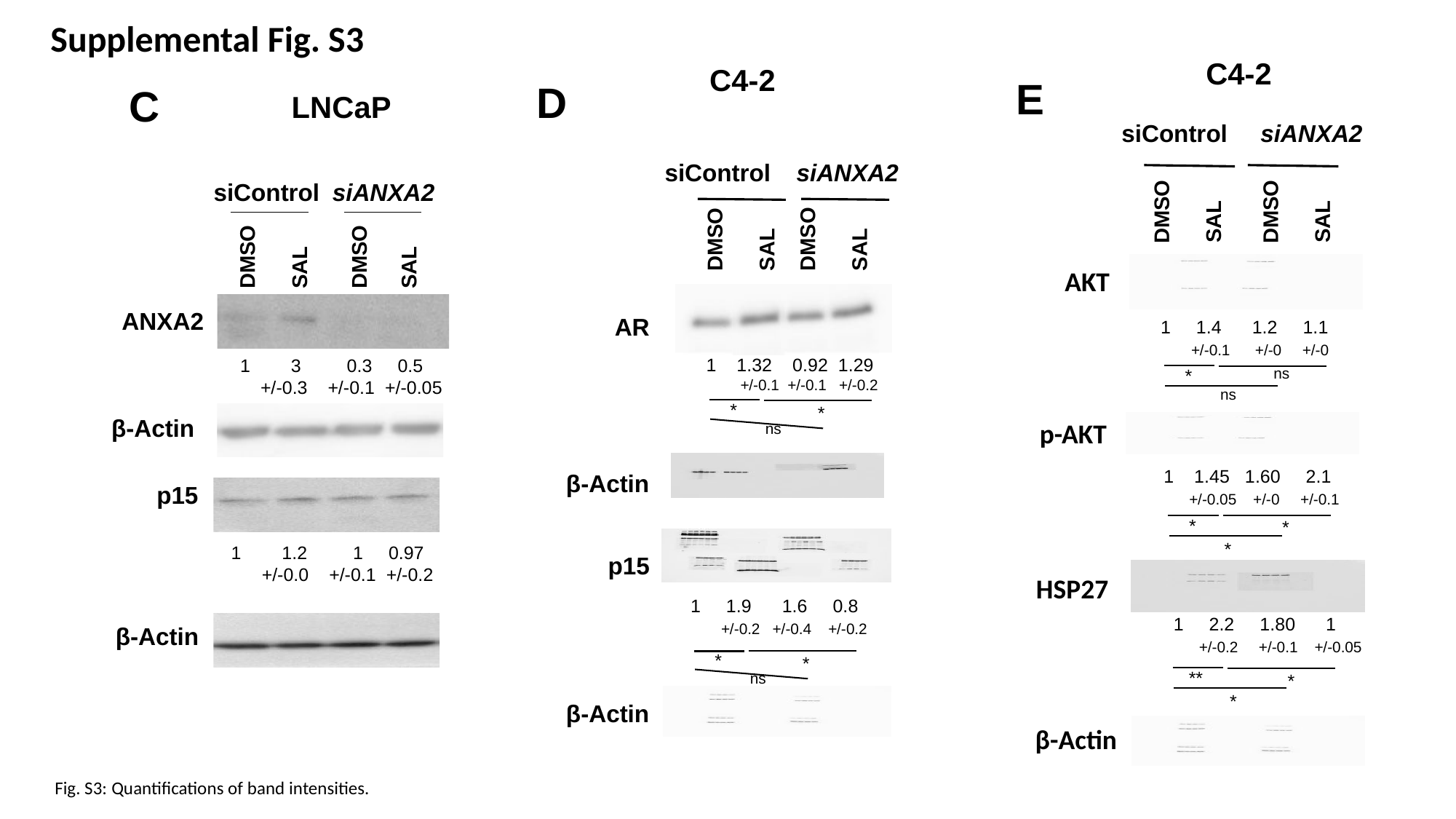

Supplemental Fig. S3
C4-2
C4-2
E
D
C
LNCaP
siControl
siANXA2
siControl
siANXA2
siControl
siANXA2
DMSO
DMSO
SAL
SAL
DMSO
DMSO
SAL
SAL
DMSO
DMSO
SAL
SAL
AKT
ANXA2
AR
 1 1.4 1.2 1.1
 +/-0.1 +/-0 +/-0
 1 1.32 0.92 1.29
 +/-0.1 +/-0.1 +/-0.2
 1 3 0.3 0.5
 +/-0.3 +/-0.1 +/-0.05
ns
*
ns
*
*
β-Actin
p-AKT
ns
 1 1.45 1.60 2.1
 +/-0.05 +/-0 +/-0.1
β-Actin
p15
*
*
*
 1 1.2 1 0.97
 +/-0.0 +/-0.1 +/-0.2
p15
HSP27
 1 1.9 1.6 0.8
 +/-0.2 +/-0.4 +/-0.2
 1 2.2 1.80 1
 +/-0.2 +/-0.1 +/-0.05
β-Actin
*
*
**
*
ns
*
β-Actin
β-Actin
Fig. S3: Quantifications of band intensities.

## Slide 4
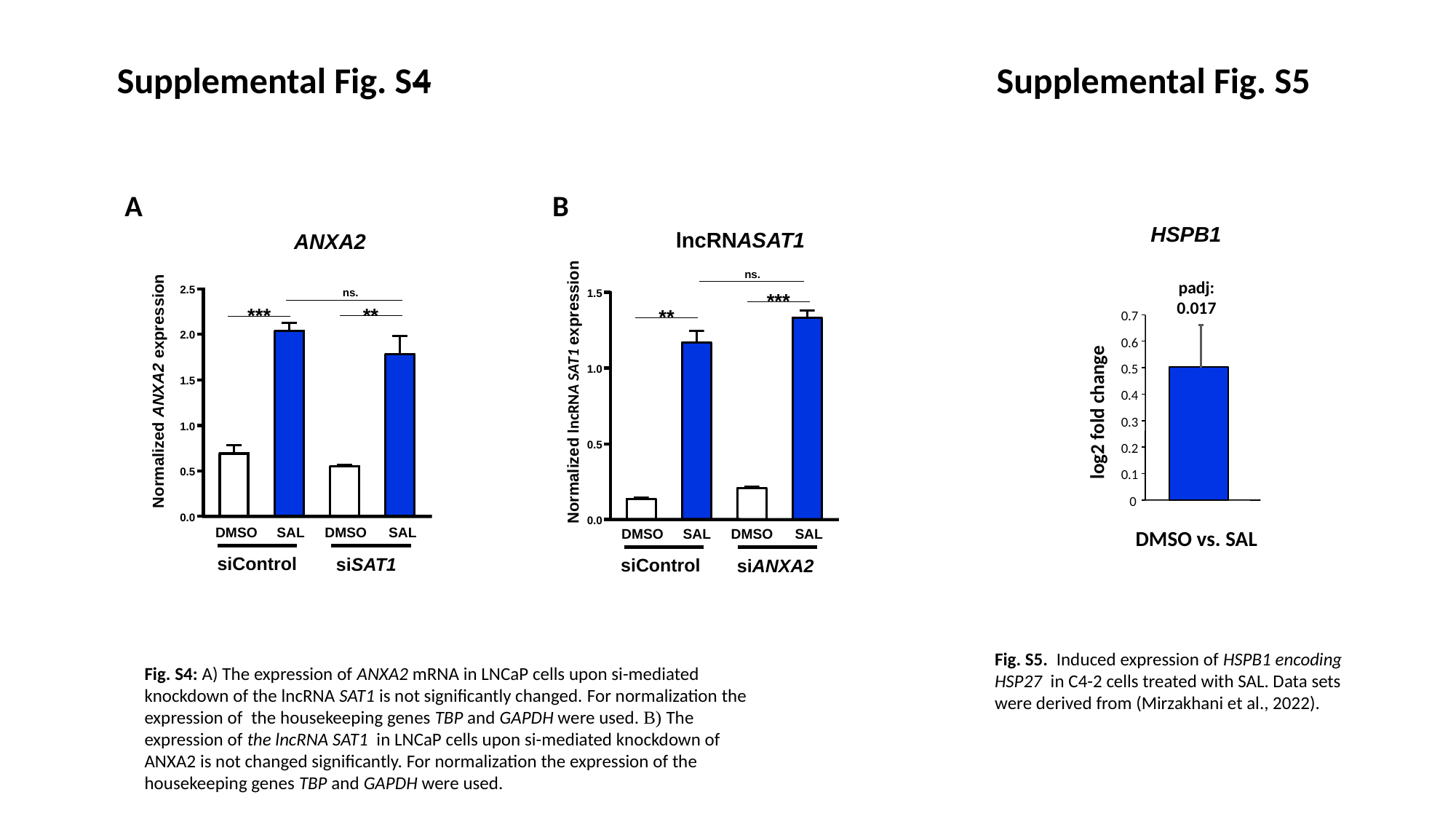

Supplemental Fig. S5
Supplemental Fig. S4
A
B
HSPB1
lncRNASAT1
ANXA2
ns.
padj:
0.017
ns.
2.5
***
1.5
***
**
**
0.7
2.0
0.6
0.5
1.0
Normalized lncRNA SAT1 expression
log2 fold change
1.5
Normalized ANXA2 expression
0.4
0.3
1.0
0.5
0.2
0.5
0.1
0
0.0
0.0
DMSO
SAL
DMSO
SAL
DMSO vs. SAL
SAL
DMSO
SAL
DMSO
siControl
siSAT1
siControl
siANXA2
Fig. S5. Induced expression of HSPB1 encoding HSP27 in C4-2 cells treated with SAL. Data sets were derived from (Mirzakhani et al., 2022).
Fig. S4: A) The expression of ANXA2 mRNA in LNCaP cells upon si-mediated knockdown of the lncRNA SAT1 is not significantly changed. For normalization the expression of the housekeeping genes TBP and GAPDH were used. B) The expression of the lncRNA SAT1 in LNCaP cells upon si-mediated knockdown of ANXA2 is not changed significantly. For normalization the expression of the housekeeping genes TBP and GAPDH were used.
